# Stress Affects a Gastrin-Releasing Peptide System in the Spinal Cord That Mediates Sexual Function: Implications for Psychogenic Erectile Dysfunction

**DOI:** 10.1371/journal.pone.0004276

**Published:** 2009-01-26

**Authors:** Hirotaka Sakamoto, Ken-Ichi Matsuda, Damian G. Zuloaga, Nobuko Nishiura, Keiko Takanami, Cynthia L. Jordan, S. Marc Breedlove, Mitsuhiro Kawata

**Affiliations:** 1 Department of Anatomy and Neurobiology, Kyoto Prefectural University of Medicine, Kawaramachi-Hirokoji, Kamigyo-ku, Kyoto, Japan; 2 Program in Neuroscience, Departments of Psychology and Zoology, Michigan State University, East Lansing, Michigan, United States of America; University of Auckland, New Zealand

## Abstract

**Background:**

Many men suffering from stress, including post-traumatic stress disorder (PTSD), report sexual dysfunction, which is traditionally treated via psychological counseling. Recently, we identified a gastrin-releasing peptide (GRP) system in the lumbar spinal cord that is a primary mediator for male reproductive functions.

**Methodology/Principal Findings:**

To ask whether an acute severe stress could alter the male specific GRP system, we used a single-prolonged stress (SPS), a putative rat model for PTSD in the present study. Exposure of SPS to male rats decreases both the local content and axonal distribution of GRP in the lower lumbar spinal cord and results in an attenuation of penile reflexes *in vivo*. Remarkably, pharmacological stimulation of GRP receptors restores penile reflexes in SPS-exposed males, and induces spontaneous ejaculation in a dose-dependent manner. Furthermore, although the level of plasma testosterone is normal 7 days after SPS exposure, we found a significant decrease in the expression of androgen receptor protein in this spinal center.

**Conclusions/Significance:**

We conclude that the spinal GRP system appears to be a stress-vulnerable center for male reproductive functions, which may provide new insight into a clinical target for the treatment of erectile dysfunction triggered by stress and psychiatric disorders.

## Introduction

Post-traumatic stress disorder (PTSD) is a psychiatric disorder involving long-lasting symptoms that may occur after exposure to a life-threatening traumatic event, and is characterized by intrusive memories (flashbacks), a hyperarousal state and avoidance of stimuli associated with the trauma [1]. Clinical data have indicated increased rates of sexual dysfunction, including erection and ejaculation difficulties in patients with PTSD [2]–[4]. Most combat veterans with PTSD experience clinically relevant sexual difficulties and 69% have erectile dysfunction (ED) [5]. Although erections are clearly androgen-dependent, as evidenced by a marked reduction in the frequency, amplitude, and rigidity of erections in men with hypogonadism [6], little is known about the role of androgen-dependent neuropathy within the central nervous system in the development of psychogenic ED. Previous studies of stress and the hypothalamic-pituitary-gonadal axis have indicated that circulating testosterone (T) fluctuates in response to physical and psychological stress [7]–[9]. As opposed to other stress-related diseases, there is evidence that plasma or serum T levels do not change in combat-related PTSD patients [10] or in refugees suffering from PTSD [11].

**Figure 1:**
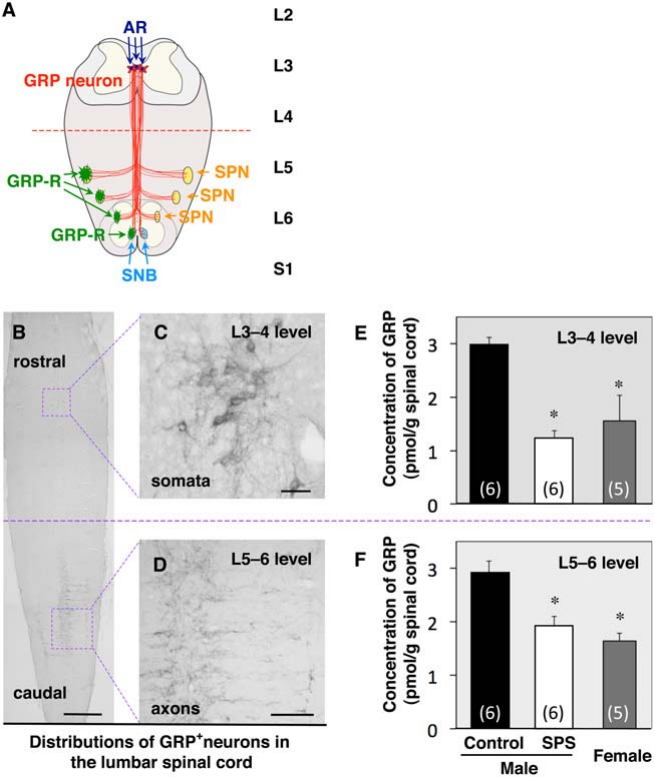
Sex difference and stress-response of GRP spinal neurons. (A) Schematic drawing summarizing the GRP system in the lumbar spinal cord that controls male reproductive functions [20]. Using a competitive ELISA for GRP, we quantified the local contents of GRP in two separate regions of the lumbar spinal cord by dividing the lumbar spinal cord into the upper (L3–4; somal region of GRP neurons; A–C) and lower (L5–6; axonal region of GRP neurons; A, B, D) spinal regions. Both in the upper and lower lumbar spinal cord, the concentrations of GRP in control males was greater than that in females (E, F). Seven days after SPS exposure, in males, GRP was significantly reduced in both the upper and lower lumbar spinal cord (E, F). *P<0.05 compared with control males. Scale bars, 1 mm (B); 100 µm (C); 200 µm (D). AR, androgen receptor; GRP-R, GRP receptor; SNB, spinal nucleus of the bulbocavernosus; SPN, sacral parasympathetic nucleus.

Gastrin-releasing peptide (GRP), a member of the bombesin-like peptide family first discovered in the skin of the frog *Bombina bombina* [12], [13], is distributed widely in the central nervous system and gastrointestinal tract of mammals [14]. GRP plays a role in many physiological processes, including itch [15], circadian rhythms [16], food intake [17] and anxiety [18]. Truitt and Coolen [19] reported that a population of neurons in the upper lumbar spinal cord acts as a ‘spinal ejaculation generator’, because a toxin treatment that selectively lesion galanin-expressing neurons there eliminates ejaculation in rats. Recently, we demonstrated that neurons within the ‘ejaculation generator’ in the upper lumbar spinal cord project axons containing GRP to the lower lumbar and upper sacral spinal cord, innervating autonomic and somatic neural regions known to control erection and ejaculation [20] (see Fig. 1A). All these target neurons express the specific receptors for GRP (GRP-R). Pharmacological stimulation of GRP-Rs systemically restores penile reflexes and ejaculation rate in castrated male rats, and antagonistic blockage of GRP-Rs *via* intrathecal catheters to this spinal region significantly attenuates penile reflexes and ejaculation rate in normal male rats [20].

In the present study, we examined whether an acute severe stress could alter the male specific GRP system. Here we show that exposure to a single-prolonged stress (SPS), a putative rat model for PTSD [21], decreases both the local content and axonal distribution of GRP in the lumbar spinal cord and results in an attenuation of penile reflexes *in vivo* more than a week later. Remarkably, administration of GRP agonist restores penile reflexes in SPS-exposed males, and also induces spontaneous ejaculation in a dose-dependent manner. In the SPS model, the stress-induced effects on the rat GRP system are not due to any detectable differences in circulating levels of androgens or corticosteroids. Our data suggest that pharmacological targeting of the spinal GRP system might relieve ED in men suffering PTSD.

## Results

### Effects of SPS on the GRP content in the lumbar spinal cord

Using a competitive enzyme-linked immunosorbent assay (ELISA) specific for GRP, we quantified the local contents of GRP in two separate regions of the lumbar spinal cord by dividing the lumbar spinal cord into upper (L3–4; somal region of GRP neurons; Fig. 1A–C) and lower (L5–6; axonal region of GRP neurons; Fig. 1A, B, D) spinal regions. In both the upper (somal) and lower (axonal) lumbar spinal cord, concentrations of GRP in control males were much higher than in females (L3–4, *F_2,14_* = 7.71, *P = 0.015; L5–6, *F_2,14_* = 7.42, *P = 0.002) (Fig. 1E, F). Seven days after SPS exposure, in males, GRP was significantly decreased in both the upper (*P = 0.002) and lower (*P = 0.005) lumbar spinal cord (Fig. 1E, F). In fact, SPS-exposed males were not significantly different from females in spinal GRP content (L3–4, *P* = 0.432; L5–6, *P* = 0.57) (Fig. 1E, F).

### Effects of SPS on the distribution of GRP in the lumbar spinal cord

**Figure 2:**
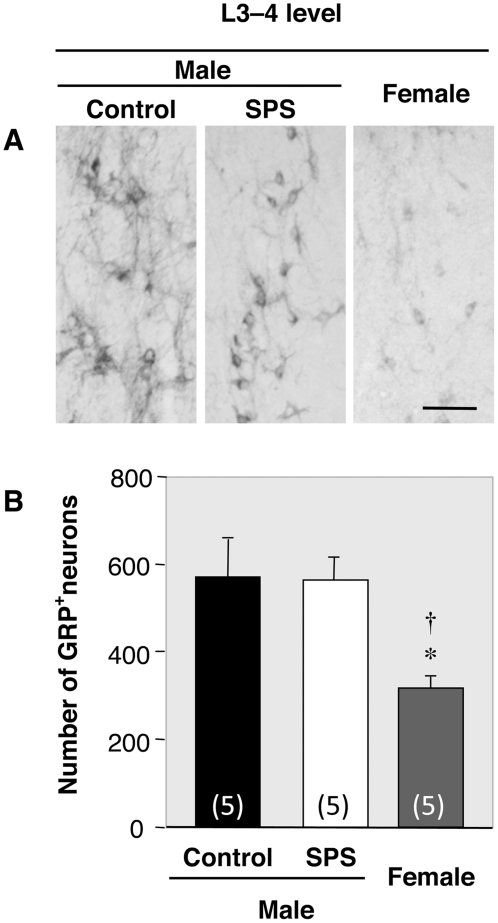
Stress-response of the GRP system in the upper lumbar spinal cord. (A, B) The number of GRP-immunoreactive neurons was greater in control males than in females in the upper lumbar spinal cord (L3–4). In contrast to overall concentrations of GRP, no significant difference between control and SPS-exposed males was observed in the number of GRP-immunoreactive neurons (B). However, the density of immunoreactive dendrites was decreased (A). Scale bar, 100 µm.

**Figure 3:**
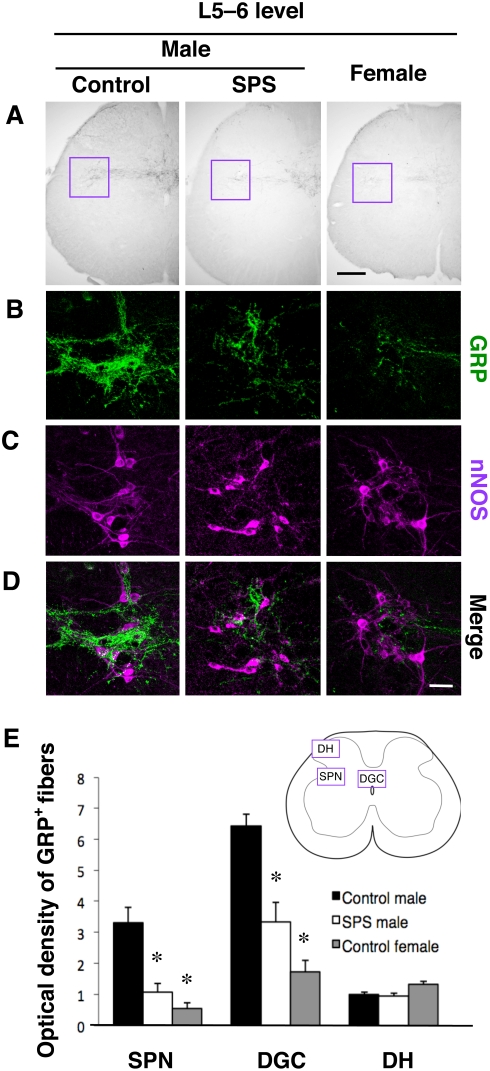
Stress-response of the GRP system in the lower lumbar spinal cord. (A–D) ICC reveals a sexual dimorphism in GRP-immunoreactive fiber distribution in lower lumbar spinal cord autonomic nuclei, as males have more GRP-immunoreactive fibers in the SPN (magenta inset) and the DGC than do females. SPS-exposure decreased the distribution of GRP-immunoreactive fibers (B), but not nNOS expression (C), to a level intermediate between control males and females (E). nNOS serves as a marker for autonomic preganglionic neurons, and double ICC reveals close appositions of GRP containing fibers with the cell bodies and proximate dendrites of nNOS-immunoreactive neurons in the SPN (D). GRP-immunoreactive fibers in a non-autonomic region of the spinal cord, the DH, are equivalent in males, females and SPS-exposed males (E). *P<0.01 compared with control males. Scale bars, 200 µm (A); 50 µm (D).

We next used immunocytochemistry (ICC) to examine the sexually dimorphic expression and stress response of GRP in the lumbar spinal cord. As expected, the number of GRP neurons in the upper lumbar spinal cord (L3–4) was much higher in control males than in females (*F_2,12_* = 7.28, *P* = 0.008) (Fig. 2A, B). In contrast to total concentration of GRP, no significant difference in the number of GRP-labeled neurons was seen between control and SPS-exposed males (*P* = 0.83) (Fig. 2B), although the intensity of immunoreactive dendrites (and/or axons) was decreased (Fig. 2A). In the lower lumbar spinal cord, GRP-containing fibers, which are distributed to autonomic centers [sacral parasympathetic nucleus (SPN)] known to regulate sexual reflexes in males, were much more prominent in control males than in females (Fig. 3A). Furthermore, GRP-containing fibers were decreased in the SPN and dorsal gray commissure (DGC) of SPS-exposed males compared to control males, but not in the dorsal horn (DH), which presumably processes non-autonomic sensory stimuli such as itch [15] (see Fig. 3A). Additionally, double ICC with GRP and neuronal nitric oxide synthase (nNOS) confirmed that GRP-containing fibers surround neurons that express nNOS (Fig. 3B–D), a marker for autonomic preganglionic neurons. Mirroring the results of ELISA for GRP, quantitative analysis of the optical density (OD) of GRP-immunoreactivity in the lower lumbar spinal cord (L5–6) confirmed that control males displayed a significantly greater OD than SPS-exposed males and females in both the SPN (*F_2,12_* = 42.21, *P*<0.001 *vs.* SPS males, *P*<0.001 *vs.* females) and DGC (*F_2,12_* = 53.95, *P*<0.001 *vs.* SPS males, *P*<0.001 *vs.* females) (Fig. 3E). GRP-containing fibers in a non-autonomic region of the spinal cord, the DH, were equivalent in control males, SPS-exposed males and control females (*F_2,12_* = 0.81) (Fig. 3E).

### SPS reduces penile reflexes, and systemic GRP treatment reinstates penile reflexes in SPS-exposed males

**Figure 4:**
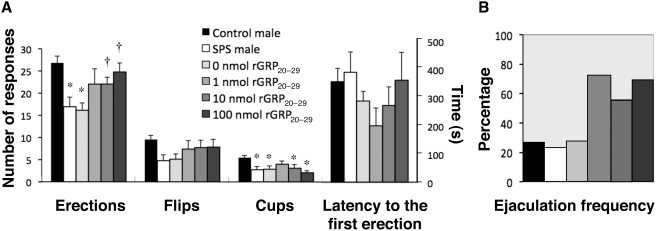
Stress and GRP involvement in male sexual reflexes. (A) SPS-exposure in male rats reduces spinal reflexes of the penis, including simple erections and cup-like flaring erections of the distal glans. Systemic treatment of SPS-exposed males with a specific agonist for GRP-R (rGRP_20–29_) restores erections and appears to affect cups and flips. (B) Spontaneous ejaculations during tests for penile reflexes are also increased by systemic rGRP_20–29_ treatment in a dose-dependent manner. *P<0.05 compared with control males. †*P*<0.05 compared to SPS-exposed males. Data are presented as means±s.e.m. (A); and as % (B).

Because SPS exposure decreased both the content and expression of GRP in the lumbar spinal cord, we administered systemic GRP agonist *in vivo* to evaluate male reproductive function, as assessed by penile reflexes, after SPS exposure. Seven days after SPS exposure males showed significantly reduced spinal reflexes of the penis, including simple erections and cup-like flaring erections of the distal glans (*n* = 9, erections, *F_5,40_* = 5.63, *P<0.001, cups, *F_5,40_* = 5.05, *P<0.001), and tended to reduce dorsal flips of the penis (*n* = 9, flips; *F_5,40_* = 1.55) (Fig. 4A). We hypothesized that GRP administration might enhance penile reflexes in SPS-exposed males. Strikingly, in SPS-exposed male rats, systemic treatment with the rat homologue of GRP-R agonists (rGRP_20–29_) restored penile erections in a dose-dependent manner (†*P*<0.003), without affecting flips and cups (Fig. 4A). Cups and flips of the penis are mediated by somatic motoneurons innervating striated muscles of the penis [22], while erections are mediated by autonomic centers, so the GRP-R agonists appeared to affect only autonomic components of sexual response in stressed males. Furthermore, the rGRP_20–29_ agents were particularly effective in restoring ejaculation, which is also autonomically mediated, resulting in a greater frequency of ejaculation not only in SPS-exposed males but also in unstressed control males (Fig. 4B). On the other hand, the latency to the first erection did not differ across groups in this study (*F_5,40_* = 1.65) (Fig. 4A).

### Effects of SPS on the expression of AR and ERα in the spinal center

**Figure 5:**
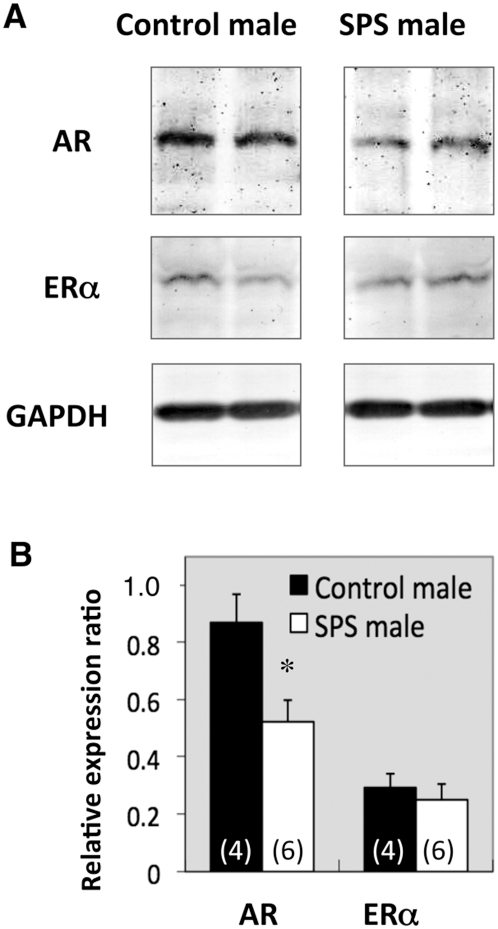
Stress affects the expression of AR and ERα protein in the upper lumbar spinal cord after SPS exposure. Representative Western immunoblot results are shown in (A). The calculated ODs of the protein bands corresponding to AR and ERα protein were normalized to each GAPDH OD and expressed as a ratio (B). The expression of AR, but not ERα, protein in the upper lumbar spinal cord (L3–4) was significantly decreased 7 days after SPS exposure (B). *P<0.05 compared with control males.

**Figure 6:**
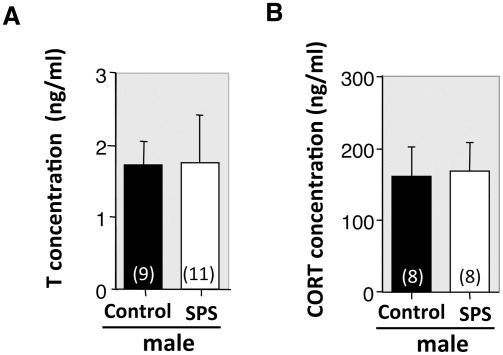
Stress does not have a prolonged effect on circulating steroid hormones. Plasma concentrations of T (A) and CORT (B) in male rats were not significantly different 7 days after SPS exposure.

We recently demonstrated that nearly all GRP neurons in the lumbar spinal cord express androgen receptor (AR), but not estrogen receptor alpha (ERα) [20]. Therefore, we used Western blotting to investigate the protein level expression of AR and ERα in spinal GRP neurons after SPS. In the lanes that were loaded with lysates of the upper lumbar spinal cords (L3–4), a single immunoreactive band was detected, corresponding to AR (∼110 kDa) and ERα (∼66 kDa) (Fig. 5A), respectively. For densitometric analyses, we calculated the expression levels of AR and ERα by dividing these values with that of glyceraldehyde-3-phosphate dehydrogenase (GAPDH), as the internal control. We found expression of AR protein in the upper lumbar spinal cord (L3–4) was significantly decreased 7 days after SPS exposure (*P = 0.023) (Fig. 5B). On the other hand, there was no effect of SPS on the expression of ERα protein (*P* = 0.57) (Fig. 5B), nor was there a significant difference in the expression of AR in the lower lumbar spinal cord (L5–6) after SPS exposure (*P* = 0.31) (data not shown). In addition, we found no significant differences in plasma T and corticosterone (CORT) levels of the SPS-exposed and control rats (Fig. 6A, B).

## Discussion

PTSD has been found to affect emotional and social functioning [1], however, a reliable animal model for PTSD has not yet been established. Recently, a number of rat studies have indicated that SPS successfully reproduces many neuroendocrine and behavioral characteristics of PTSD, including enhanced hypothalamo-pituitary-adrenal negative feedback, exaggerated acoustic startle response, and increased contextual freezing 7 days after SPS exposure [21], [23]–[25]. Taken together, these findings indicate that SPS induces time-dependent sensitization, which resembles the developmental course of PTSD and represents an animal model for PTSD. It has long been established that ED is a multi-factorial dysfunction, and some treatments for ED patients have been developed with a focus mainly on therapeutics for penile vasculopathy. Recently, we demonstrated that a system of neurons in the upper lumbar spinal cord utilize a specific peptide, GRP, to drive lower spinal centers that coordinate male reproductive functions such as erection and ejaculation [20] (see Fig. 1A). The present data show that SPS significantly attenuates penile reflexes in rats. Thus in addition to the other features of PTSD in humans that have been previously described in rats following SPS, we find the rats also display male reproductive dysfunctions after SPS. SPS also attenuated two aspects of the GRP system, the expression of GRP *per se* and the expression of ARs that regulate penile reflexes. Furthermore, pharmacological stimulation of GRP receptors restores male reproductive function in male rats exposed to SPS. These data suggest that the effects of SPS to inhibit male reproductive function are mediated, at least in part, by effects on the spinal GRP system. To the best of our knowledge, this is the first demonstration of a center for male reproductive function in the spinal cord that can be altered in the expression and/or distribution of protein by exposure to stress. Since a significant reduction in the frequency of penile reflexes was observed in SPS-exposed males, this may also serve as a viable paradigm to study the pathophysiology of psychogenic ED in PTSD or other disorders.

Considerable evidence suggests that GRP has complex molecular interactions in various tissues. GRP stimulates neuroblastoma growth and the expression of angiogenic markers, platelet endothelial cell adhesion molecule-1 and vascular endothelial growth factor expressions [26]. Moreover, GRP activates nuclear factor kappa B-dependent pathway to modulate the expressions of interleukin-8 and vascular endothelial growth factor in prostate cancer cells [27]. Thus, GRP regulates the expression of many genes to affect cellular function both in the neural and non-neural cells. When stress suppresses GRP expression in the spinal cord, it presumably involves a cascade of other stress-response genes, both upstream and downstream from GRP itself.

Castration of adult male rats significantly reduced the expression of GRP in the lumbar spinal cord, and this reduction was averted by androgen replacement [20]. Additionally, the spinal GRP system is completely feminine in XY rats with a dysfunctional AR gene [20]. Thus T and AR are both necessary to maintain normal GRP expression in the lumbar spinal cord [20]. Men with major depression have decreased T levels and negative correlation between T levels and the severity of depression has been observed [8], [28]. Similarly, exposure to chronic restraint stress in rats, a rodent model of depression, decreases plasma levels of T [7], [29], suggesting a relationship with male reproductive dysfunctions. Our results with SPS in rats are consistent with previous reports of normal plasma T levels in combat-related PTSD patients [10] and in refugees suffering from PTSD [11]. However, cerebrospinal fluid T levels were lower in combat-related PTSD patients as compared with normal controls [10]. Although the level of plasma T is normal 7 days after SPS exposure, we found a significant decrease in the expression of AR but not ERα protein in the upper lumbar spinal cord. In control males, nearly every GRP-immunoreactive neuron in the upper lumbar spinal cord also contains AR [20]. Taken together, these results suggest that the decline of AR expression in the upper lumbar spinal cord may be a link in the attenuation of the GRP system after SPS exposure, and consequently contributes to the appearance of sexual dysfunctions, including erection and ejaculation difficulties. Future study is required to clarify the mechanism(s) involved in down-regulating AR expression in the spinal sexual center in response to the severe psychological stress.

Stress responses are typically mediated through glucocorticoid receptors in the central nervous system [30], [31]. Although plasma levels of CORT in patients with major depression are high, most have demonstrated subnormal CORT levels in the plasma of humans with PTSD, despite the increased release of corticotrophin releasing factor, suggesting an exaggerated hypothalamo-pituitary-adrenal axis negative feedback [32], [33]. Similarly, in rats, we found plasma CORT level as well as T were still in the physiological range 7 days after SPS (see Fig. 6). Furthermore, we see no difference in the expressions of glucocorticoid receptor protein in the upper lumbar spinal cord (L3–4) of SPS-exposed males as revealed by Western blotting (our unpublished observation). Thus the GRP system in the rat lumbar spinal cord may be independent of CORT after SPS exposure.

In the present study, we did not use the GRP antagonist RC-3095 in SPS-exposed rats because they already display few reflexes. Furthermore, installing intrathecal catheters to the lower lumbar spinal cord to deliver the antagonist, would itself act as a stressor, making it difficult to see an effect of SPS. However, we previously inserted intrathecal catheters in otherwise unstressed rats and found that RC-3095 suppresses sexual reflexes in a dose-dependent manner [20]. These studies, taken together, support the hypothesis that SPS suppresses erectile reflexes by affecting the spinal GRP system.

In summary, we found that stress significantly attenuates the spinal GRP system regulating male reproductive function, reducing expression of both GRP and the ARs mediating T effects on the system, interfering with male reproductive function. These findings suggest that the use of selective GRP-R agents may provide new avenues for the treatment of stress-related ED in men.

## Materials and Methods

### Animals

Adult male Sprague-Dawley rats were maintained in air-conditioned rooms (22±1°C) on a 12 h light/dark cycle with free access to food and drinking water. All experimental procedures were authorized by the Committee for Animal Research, Kyoto Prefectural University of Medicine, Japan and/or Michigan State University, USA.

### SPS exposure

Rats were randomly assigned to one of two experimental groups: control group and SPS group, and were housed in pairs. After an acclimation period, male rats were subjected to SPS as previously described [21], [23]-[25], [34], [35]. Males were exposed to a single session of prolonged stress consisting of restraint for 2 h in an acrylic restrainer (55×45×200 mm, NeuroScience Idea, Osaka, Japan) (10:00 to noon) followed immediately by forced swimming for 20 min in 23±1°C water. The animals were allowed to recuperate for 15 min and then were exposed to ether vapor until loss of consciousness. The animals were then returned to their home cages (2 rats per cage) and left undisturbed for 7 days. Control animals of both sexes were not subjected to any stress, and were housed in an undisturbed environment during the SPS experiments.

### ICC staining

We performed the ICC analysis according to established methods [20], [34]–[36]. The primary rabbit antiserum against GRP (1∶5,000) (Phoenix Pharmaceuticals, Burlingame, CA) was used [20]. For analysis of quantity, GRP-immunoreactive cells with clearly visible transected round nuclei were counted in the anterior part of the lumbar spinal cord (L3–4 level). To determine the density of positive GRP-immunoreactive fibers in the posterior part of the lumbar spinal cord (L5–6 level), at least ten sections per animals were analyzed using ImageJ software (ImageJ 1.36b) with a set threshold level. GRP-immunoreactive fiber pixel density was quantified as the average pixel density in three regions of each animal, the SPN, DGC and DH, and was calculated as the ratio to the density seen in the DH in control males. At least 5 animals were used in each group.

To determine the projection site of GRP-immunoreactive axons, double-immunofluorescence staining of GRP (1∶5,000 dilution) and nNOS (A-11; mouse monoclonal antibody, Santa Cruz Biotechnology, Santa Cruz, CA) (1∶8,000 dilution), a marker protein for neurons in the SPN, was performed as described previously [20].

### Behavior tests

All behavior tests were conducted between 08:30 and 13:30 h and included at least 9 animals per group. Penile reflex tests consisted of holding a rat in a supine position in a Plexiglas cylinder for a period of 25 min as described previously [20], [37]. After 5 min of adaptation in the cylinder, the rat's penile sheath was rolled back with a wooden cotton-tipped applicator to expose the glans. The occurrence of erections, cups, and flips and the latency to the first erection were recorded on an event recorder for a period of 20 min. Erections were scored when the penis became blood engorged and swollen in size, a cup involved the flaring out of the tip of the phallus to a circumference equal to or greater than the base of the glans, and a flip involved a dorsal deflection of 30° or more from resting position. The occurrence of the spontaneous ejaculation during the adaptation in the cylinder was also recorded. Animals received at least two behavior tests prior to SPS exposure as a control. Animals were administered behavior tests after a 30 min intraperitoneal injection of rat GRP_20–29_, a specific agonist for GRP-R, diluted in saline (0, 1, 10 and 100 nmol/kg body weight).

### Peptide extraction and ELISA

Peptides were extracted according to our previous methods [34], [35]. The concentration of GRP in the upper (L3–4) and lower (L5–6) lumbar spinal cord was measured by a competitive ELISA using a kit for GRP (Phoenix Pharmaceuticals) according to the manufacturer's protocol. The concentration of GRP was calculated in terms of picomoles per gram wet weight (pmol/g tissue) of each spinal cord. We included the standard curve in each experiment.

### Western blot analysis

Western blot analyses were conducted as previously described [36]. The lysates derived from L3–4 level were run on a 7.5% SDS-PAGE. After blotting, PVDF membranes were probed with anti-AR (N-20; Santa Cruz Biotechnology, 1∶500) [38], anti-ERα (MC-20; Santa Cruz Biotechnology, 1∶2,000) [39] and anti-GAPDH (6C5, abcam, Cambridge, MA, 1∶1,000). Results were quantified by densitometric analysis using ImageJ software, and were expressed as the OD for ratio to each GAPDH expression level.

### Enzyme immunoassay

Rats were intraperitoneally injected with sodium pentobarbital between 10:00 and 11:00 h before cage movement, and they were then decapitated and blood samples were collected at 30 min after the injection. The samples were centrifuged immediately at 4,000×*g* at 4°C, and blood plasma was stored at −80°C until assay. Plasma concentrations of T and CORT were measured using specific enzyme immunoassay kits (Cayman Chemical, Ann Arbor, MI) as described previously [20], [35].

### Statistical analysis

Data are expressed as the standard error of the mean (s.e.m.). We derived *P* values for Figs. 1E, F; and 2B using a one-way analysis of variance (ANOVA), for Fig. 3E using a two-way ANOVA, and for Fig. 4A using an ANOVA with repeated measures. When significant main effects were found, *post hoc* Bonferroni tests were performed. Significance in Figs. 5B and 6A, B was tested with an unpaired Student's *t*-test. Differences were considered significant if *P*<0.05.

## References

[1] Pitman RK (1997). Overview of biological themes in PTSD.. Ann N Y Acad Sci.

[2] Cosgrove DJ, Gordon Z, Bernie JE, Hami S, Montoya D (2002). Sexual dysfunction in combat veterans with post-traumatic stress disorder.. Urology.

[3] Kaplan HS (1989). Post-traumatic stress syndrome and sexual dysfunction.. J Sex Marital Ther.

[4] Kaplan HS (1988). Anxiety and sexual dysfunction.. J Clin Psychiatry.

[5] Letourneau EJ, Schewe PA, Frueh BC (1997). Preliminary evaluation of sexual problems in combat veterans with PTSD.. J Trauma Stress.

[6] Rajfer J (2000). Relationship between testosterone and erectile dysfunction.. Rev Urol.

[7] Retana-Marquez S, Bonilla-Jaime H, Vazquez-Palacios G, Martinez-Garcia R, Velazquez-Moctezuma J (2003). Changes in masculine sexual behavior, corticosterone and testosterone in response to acute and chronic stress in male rats.. Horm Behav.

[8] Mason JW, Giller EL, Kosten TR (1988). Serum testosterone differences between patients with schizophrenia and those with affective disorder.. Biol Psychiatry.

[9] Kreuz LE, Rose RM, Jennings JR (1972). Suppression of plasma testosterone levels and psychological stress. A longitudinal study of young men in Officer Candidate School.. Arch Gen Psychiatry.

[10] Mulchahey JJ, Ekhator NN, Zhang H, Kasckow JW, Baker DG (2001). Cerebrospinal fluid and plasma testosterone levels in post-traumatic stress disorder and tobacco dependence.. Psychoneuroendocrinology.

[11] Bauer M, Priebe S, Graf KJ, Kurten I, Baumgartner A (1994). Psychological and endocrine abnormalities in refugees from East Germany: Part II. Serum levels of cortisol, prolactin, luteinizing hormone, follicle stimulating hormone, and testosterone.. Psychiatry Res.

[12] Anastasi A, Erspamer V, Bucci M (1971). Isolation and structure of bombesin and alytesin, 2 analogous active peptides from the skin of the European amphibians Bombina and Alytes.. Experientia.

[13] McDonald TJ, Jornvall H, Nilsson G, Vagne M, Ghatei M (1979). Characterization of a gastrin releasing peptide from porcine non-antral gastric tissue.. Biochem Biophys Res Commun.

[14] Panula P, Nieminen O, Falkenberg M, Auvinen S (1988). Localization and development of bombesin/GRP-like immunoreactivity in the rat central nervous system.. Ann N Y Acad Sci.

[15] Sun YG, Chen ZF (2007). A gastrin-releasing peptide receptor mediates the itch sensation in the spinal cord.. Nature.

[16] Shinohara K, Tominaga K, Isobe Y, Inouye ST (1993). Photic regulation of peptides located in the ventrolateral subdivision of the suprachiasmatic nucleus of the rat: daily variations of vasoactive intestinal polypeptide, gastrin-releasing peptide, and neuropeptide Y.. J Neurosci.

[17] Ladenheim EE, Taylor JE, Coy DH, Moore KA, Moran TH (1996). Hindbrain GRP receptor blockade antagonizes feeding suppression by peripherally administered GRP.. Am J Physiol.

[18] Merali Z, Bedard T, Andrews N, Davis B, McKnight AT (2006). Bombesin receptors as a novel anti-anxiety therapeutic target: BB1 receptor actions on anxiety through alterations of serotonin activity.. J Neurosci.

[19] Truitt WA, Coolen LM (2002). Identification of a potential ejaculation generator in the spinal cord.. Science.

[20] Sakamoto H, Matsuda K-I, Zuloaga DG, Hongu H, Wada E (2008). Sexually dimorphic gastrin releasing peptide system in the spinal cord controls male reproductive functions.. Nat Neurosci.

[21] Liberzon I, Krstov M, Young EA (1997). Stress-restress: effects on ACTH and fast feedback.. Psychoneuroendocrinology.

[22] Sachs BD (1982). Role of striated penile muscles in penile reflexes, copulation, and induction of pregnancy in the rat.. J Reprod Fertil.

[23] Khan S, Liberzon I (2004). Topiramate attenuates exaggerated acoustic startle in an animal model of PTSD.. Psychopharmacology (Berl).

[24] Kohda K, Harada K, Kato K, Hoshino A, Motohashi J (2007). Glucocorticoid receptor activation is involved in producing abnormal phenotypes of single-prolonged stress rats: a putative post-traumatic stress disorder model.. Neuroscience.

[25] Iwamoto Y, Morinobu S, Takahashi T, Yamawaki S (2007). Single prolonged stress increases contextual freezing and the expression of glycine transporter 1 and vesicle-associated membrane protein 2 mRNA in the hippocampus of rats.. Prog Neuropsychopharmacol Biol Psychiatry.

[26] Kang J, Ishola TA, Baregamian N, Mourot JM, Rychahou PG (2007). Bombesin induces angiogenesis and neuroblastoma growth.. Cancer Lett.

[27] Levine L, Lucci JA, Pazdrak B, Cheng JZ, Guo YS (2003). Bombesin stimulates nuclear factor kappa B activation and expression of proangiogenic factors in prostate cancer cells.. Cancer Res.

[28] Yesavage JA, Davidson J, Widrow L, Berger PA (1985). Plasma testosterone levels, depression, sexuality, and age.. Biol Psychiatry.

[29] Matsumoto A (2005). Testosterone prevents synaptic loss in the perineal motoneuron pool in the spinal cord in male rats exposed to chronic stress.. Stress.

[30] Chrousos GP, Kino T (2007). Glucocorticoid action networks and complex psychiatric and/or somatic disorders.. Stress.

[31] Holsboer F, Grasser A, Friess E, Wiedemann K (1994). Steroid effects on central neurons and implications for psychiatric and neurological disorders.. Ann N Y Acad Sci.

[32] Yehuda R (1997). Sensitization of the hypothalamic-pituitary-adrenal axis in posttraumatic stress disorder.. Ann N Y Acad Sci.

[33] Yehuda R (2002). Post-traumatic stress disorder.. N Engl J Med.

[34] Cui H, Sakamoto H, Higashi S, Kawata M (2008). Effects of single-prolonged stress on neurons and their afferent inputs in the amygdala.. Neuroscience.

[35] Yoshii T, Sakamoto H, Kawasaki M, Ozawa H, Ueta Y (2008). The single-prolonged stress paradigm alters both the morphology and stress response of magnocellular vasopressin neurons.. Neuroscience.

[36] Sakamoto H, Matsuda K-I, Hosokawa K, Nishi M, Morris JF (2007). Expression of G protein-coupled receptor-30, a G protein-coupled membrane estrogen receptor, in oxytocin neurons of the rat paraventricular and supraoptic nuclei.. Endocrinology.

[37] Monaghan EP, Breedlove SM (1992). The role of the bulbocavernosus in penile reflex behavior in rats.. Brain Res.

[38] Chen CD, Welsbie DS, Tran C, Baek SH, Chen R (2004). Molecular determinants of resistance to antiandrogen therapy.. Nat Med.

[39] Cheng G, Weihua Z, Warner M, Gustafsson JA (2004). Estrogen receptors ER alpha and ER beta in proliferation in the rodent mammary gland.. Proc Natl Acad Sci U S A.

